# Ossification of the Pulp of the Teeth

**Published:** 1851-07

**Authors:** W. W. Codman

**Affiliations:** Boston


### OSSIFICATION OF THE PULP OF THE TEETH.

*To the Editor of the Boston Medical and Surgical Journal.*

Dear Sir,—Seeing my name in an article copied by you in the Dental Journal, I beg leave to make some explanations in regard to it. Dr. Harris, editor of that Journal, seems to have forgotten the most important part of my statement, which was that I waited until the ossification had taken place before I filled the tooth. My principle is, to excite ossification, as surgeons sometimes do to fractured bones when indolent. And I have succeeded in doing it, in many cases, where the dental pulp is healthy, even though wounded. By cleansing the cavity, as if for filling, then protecting it with cotton from the air, and occasionally removing the cotton and lightly re-scraping the bone, a deposit, in time, will take place nearly as hard as enamel, when the tooth can be filled and retain its vitality. Twelve years' experience in this operation has proved this fact to me, that, under favorable circumstances, it can be done.

Boston, Dec. 11, 1850. W. W. Codman.

